# The effect of continuous bilateral parasternal block with lidocaine on patient-controlled analgesia opioid requirement and recovery after open heart surgery: a double-blind randomised controlled trial

**DOI:** 10.1016/j.bjao.2024.100279

**Published:** 2024-04-20

**Authors:** Mark Larsson, Ulrik Sartipy, Anders Franco-Cereceda, Anders Öwall, Jan Jakobsson

**Affiliations:** 1 Department of Molecular Medicine and Surgery, Karolinska Institutet, Stockholm, Sweden; 2 Function Perioperative Medicine and Intensive Care, Section for Cardiothoracic Anaesthesia and Intensive Care, Sweden; 3 Department of Cardiothoracic Surgery, Karolinska University Hospital, Stockholm, Sweden; 4 Institution for Clinical Sciences, Karolinska Institutet at Danderyd Hospital, Stockholm, Sweden; 5 Department of Anaesthesia and Intensive Care, Danderyd Hospital, Stockholm, Sweden

**Keywords:** cardiac surgery, lidocaine, parasternal block, postoperative pain, quality of recovery, sternotomy

## Abstract

**Background:**

We hypothesised that a continuous 72-h bilateral parasternal infusion of lidocaine at 2×35 mg h^−1^ would decrease pain and the inflammatory response after sternotomy for open heart surgery, subsequently improving quality of recovery.

**Methods:**

We randomly allocated 45 participants to a 72-h bilateral parasternal infusion of lidocaine or saline commencing after wound closure. The primary outcome was the cumulative patient-controlled analgesia (PCA) morphine consumption at 72 h. Secondary outcomes included total morphine requirement, pain, peak expiratory flow, and serum interleukin-6 concentration. In addition, we used an eHealth platform for a 3-month follow-up of pain, analgesic use, and Quality of Recovery-15 scores.

**Results:**

The 72-h PCA morphine requirement was significantly lower in the lidocaine than the saline group (10 mg [inter-quartile range: 5–19 mg] and 28.2 mg [inter-quartile range: 16–42.5 mg], respectively; *P*=0.014). The total morphine requirement (including morphine administered before the start of PCA) was significantly lower at 24, 48, and 72 h. Pain was well controlled with no difference in pain scores between treatment groups. The peak expiratory flow was lower in the lidocaine group at 72 h. Interleukin-6 concentrations showed no difference at 24, 48, or 72 h. Quality of Recovery-15 scores did not differ between treatment groups at any time during the 3-month follow-up.

**Conclusions:**

After sternotomy for open heart surgery, a 72-h bilateral parasternal lidocaine infusion significantly decreased PCA and total morphine requirement. However, neither signs of decreased inflammatory response nor an improvement in recovery was seen.

**Clinical trial registration:**

EudraCT number 2018-004672-35.

Enhanced recovery protocols advocate the perioperative use of local anaesthetics as part of a multimodal opioid-sparing analgesic strategy.^1^ Local anaesthetics can be administered for neuraxial and peripheral nerve blocks, and local wound infiltration. Thoracic epidural anaesthesia is effective for analgesia after open cardiac surgery,^2^ ^3^ but is not widely used. Bilateral parasternal blocks and sternal wound infiltration have both been recommended^4^ for use after sternotomy. The insertion of one or two (bilateral) catheters for continuous local anaesthetic infusion can provide a prolonged block and is therefore an attractive analgesic method during the first few days after surgery. Previous trials that infused bupivacaine or ropivacaine^5^, ^6^, ^7^, ^8^, ^9^, ^10^, ^11^, ^12^ reported various results, but the overall evidence indicates that a peripheral nerve block of the sternum reduces pain and opioid requirement.^13^ However, the type, concentration, and rate of local anaesthetic infusion influence the efficacy of the block and need further study.^13^ In addition, whether the reduced opioid requirement results in an improved recovery is unknown.

Any nerve block results in systemic local anaesthetic uptake, which can result in additional, systemic effects. All local anaesthetics have anti-inflammatory properties, although these may be less with ropivacaine.^14^ ^15^ Lidocaine has been demonstrated to have anti-inflammatory effects^16^ and, when given intravenously (i.v.), it has antihyperalgesic, analgesic, and antiarrhythmic properties.^17^, ^18^, ^19^ I.V. lidocaine results in improved postoperative recovery, as evidenced by an improved Quality of Recovery (QoR)-40 score.^20^

We hypothesised that a continuous 72-h bilateral parasternal block with lidocaine would decrease opioid requirements after open heart surgery. We expected a decrease in opioid-related side-effects combined with a systemic analgesic and anti-inflammatory effect of lidocaine to result in an improved recovery.

## Methods

This double-blind, randomised controlled trial compared lidocaine at 5 mg ml^−1^ with saline in a continuous 72-h bilateral parasternal infusion after sternotomy for open heart surgery. The trial was approved by the Swedish Ethical Review Authority (registration number 2019–05120) and the Swedish Medical Products Agency (registration number 5.1-2019-78659). It was registered as a clinical trial (EudraCT number 2018-004672-35, https://www.clinicaltrialsregister.eu/ctr-search/trial/2018-004672-35/SE). The trial protocol was published before enrolment was concluded.^21^

### Recruitment

Men and non-pregnant women with an American Society of Anesthesiologists physical status classification (ASA) of 2–3 scheduled for elective open heart surgery through a sternotomy at the Karolinska University Hospital were considered for enrolment. Reasons for ineligibility were emergency or redo surgery, severe left heart failure, respiratory insufficiency, advanced kidney failure, pronounced hepatic disease, allergy to local anaesthetics, psychiatric disease or any psychoactive medication, cognitive disturbance, or inability to understand written or oral instructions, and a history of chronic pain or chronic pain medication. Exclusion criteria after enrolment were deep hypothermia during surgery and the need for tracheal intubation to be maintained for >4 h after surgery. Eligible patients received oral and written information about the trial.

### Randomisation and blinding

Treatment was randomised in a 1:1 fashion in blocks of eight, except for the last block of five. A detailed description of the randomisation process is available in the Supplementary material. The trial participants, investigators, attending surgeons, anaesthesiologist, and nurse anaesthetist, and the nurses in the recovery room and surgical ward, were blinded to allocation.

### Intervention

A detailed description of the intervention has been previously published^21^ and is also available in the Supplementary material. After wound closure at the end of surgery, a multi-hole 19-cm silver-coated catheter (ON-Q Soaker; Avanos Medical, Alpharetta, GA, USA) was inserted on either side of the sternum, under the pectoral muscle and over the costosternal margin.^8^ Depending on group allocation, a 20-ml bolus of either lidocaine 5 mg ml^−1^ (LIDO group) or saline 9 mg ml^−1^ (0.9%) (SAL group) was administered through each catheter. Thereafter, an elastomeric pump that contained the allocated treatment provided an infusion of 7 ml h^−1^ to each catheter. After tracheal extubation, and when the patient was considered to have more than mild pain, the attending nurse administered i.v. morphine, ketorolac, or clonidine. When the participant was alert and experienced mild pain at most (numerical rating scale [NRS] score of ≤3), a patient-controlled analgesia (PCA) pump for i.v. morphine administration was connected (1-mg bolus, 6-min lockout time, and maximum of 30 mg every 4 h).

All participants received care according to standard departmental practice except for the allocated treatment. Pain was controlled by use of the morphine PCA pump, in addition to oral paracetamol 1 g four times daily. As rescue analgesic treatment, i.v. or oral clonidine 75 μg three to four times daily, i.v. ketorolac 15 mg, or oral naproxen 250–500 mg twice daily could be given. Prophylaxis for postoperative nausea and vomiting (PONV) consisted of betamethasone 4 mg and ondansetron 4 mg.

At 72 h, the parasternal catheters were removed, the PCA pump was stopped, and an oral opioid regimen consisting of slow-release oxycodone at a dose equivalent to the PCA morphine requirement of the previous 24 h was initiated. Oral paracetamol and, if started earlier, naproxen were continued.

### Outcome and safety measurements

The primary outcome was the cumulative administration of i.v. PCA morphine at 72 h. The total morphine dose after surgery, defined as the sum of nurse-administered morphine before starting the PCA pump and PCA-administered morphine, was a secondary outcome. Further secondary outcomes were pain, quality of recovery, peak expiratory flow (PEF), serum interleukin (IL)-6 concentration, and oxycodone requirement from 2 weeks until 3 months after surgery. From the evening before surgery, and at pre-determined time points, automated push notices to a participant's mobile phone prompted them to access an eHealth platform and complete questionnaires on pain (NRS score), PONV, recovery (QoR-15 scale), and analgesic use.

Pain after surgery was assessed by NRS at rest and after two deep breaths three times daily (7 am, 2 pm, and 8 pm) until postoperative day (POD) 3, and thereafter at 2 weeks, and at 1, 2, and 3 months after surgery. The instructions to the study participants were that NRS 0 represented no pain, whereas NRS 1–3 indicated mild, NRS 4–6 moderate, and NRS 7–10 severe pain. Quality of recovery was assessed by the QoR-15 score^22^ the evening before surgery, in the evening of POD 1, 2, and 3, and thereafter at 2 weeks, and at 1, 2, and 3 months after surgery. At the latter four time points, participants were also asked if they required any analgesic. The average of three consecutive PEF measurements before surgery and on POD 1, 2, and 3 was used for comparisons. Blood samples were drawn and analysed for the serum concentration of IL-6 before surgery and at 1, 24, 48, and 72 h.

For safety, the incidence of any of the following during the 72-h intervention was monitored: PONV; sedation (alert *vs* non-alert); arrhythmia (more than single or solitary coupled supraventricular and ventricular extra beats); serum lidocaine concentration at 1, 24, 48, and 72 h; and the occurrence of any serious adverse event (SAE) until 3 months after surgery.

### Statistical analyses

Clinical follow-up data at our institution showed a 48-h morphine requirement of 67 mg (standard deviation [sd], 30 mg) after sternotomy. Morphine requirement decreased by 14 mg when patients received a bilateral parasternal block with ropivacaine at 8 mg h^−1^. In selected cases, we replaced ropivacaine for lidocaine and saw a further reduction in morphine requirement. For the sample size calculation, we assumed a parasternal block with lidocaine would at least double the morphine reduction and result in a 48-h morphine requirement of <40 mg. To identify a significant difference of at least 27 mg, assuming an sd of 30 mg for the difference (*P*<0.05), and with 80% power, would require the inclusion of 42 participants. We planned to include 45 participants. All secondary outcomes were exploratory.

Data were analysed per protocol. Safety measures were analysed until the end of follow-up. In a *post hoc* analysis, we categorised pain into mild (NRS≤3) and non-mild pain (NRS>3). Continuous normally distributed data are summarised as mean and sd, non-normally distributed data as median and inter-quartile range (IQR). Categorical variables are presented as number, frequency (percentage), or both. Depending on normality, continuous data were analysed with Student's *t*-test or Wilcoxon rank-sum test. Categorical data were analysed with Pearson's χ^2^ or Fisher's exact test. Repeated measurements were analysed with the Friedman test and Wilcoxon sign rank for individual comparisons. Statistical analysis was performed using Stata 14.2 software (StataCorp, College Station, TX, USA).

## Results

**Fig 1:**
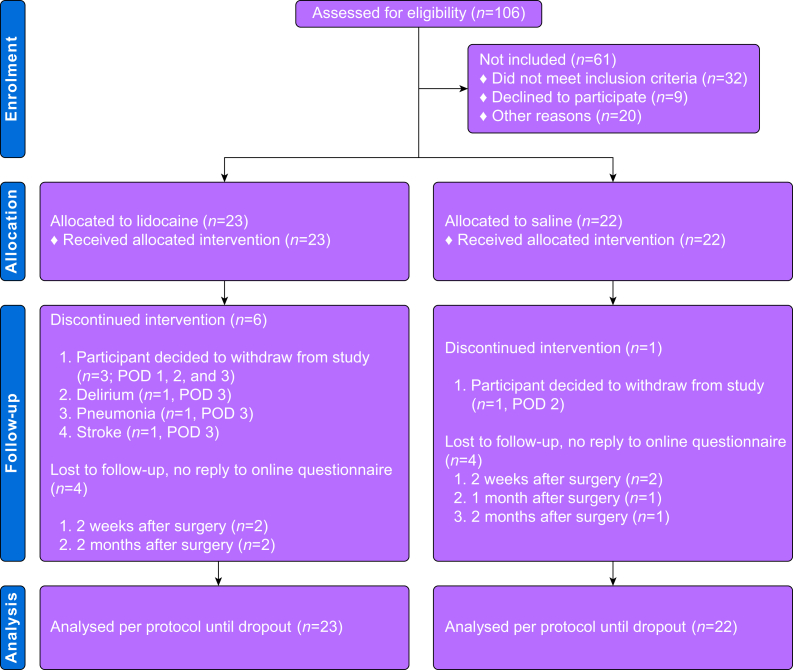
Consolidated standards of reporting trials **(**CONSORT) diagram. POD, postoperative day.

From 1 June 2021 to 21 February 2022, 106 patients were screened for participation. Sixty-one patients were ineligible, the remaining 45 patients were enrolled. After enrolment, six participants in the LIDO group and one in the SAL group discontinued the 72-h intervention. An additional two participants in the LIDO group and three in the SAL group were lost during the 3-month follow-up (Fig. 1). The return percentage of the NRS and QoR scores on the digital platform was lower for the LIDO group than the SAL group at POD 1–3 (Fig. 1 Supplementary Material).

Participants in the LIDO group had a lower BMI at baseline than those in the SAL group (25 [sd 3] and 28 [sd 6] kg m^−2^, respectively). No other differences in baseline characteristics were observed (Table 1). Neither the perioperative fentanyl dose, nor the use of paracetamol, NSAID or clonidine differed between groups (Table 2).

**Table 1:** Baseline characteristics of study participants who received a continuous parasternal block after sternotomy. Data are presented as number (%), median (inter-quartile range), or mean (standard deviation). BMI, body mass index; CABG, coronary artery bypass grafting; LIDO, lidocaine; SAL, saline. ∗*P*=0.020.

	LIDO group (*n*=23)	SAL group (*n*=22)
Sex		
Male	21 (91)	19 (86)
Female	2 (9)	3 (14)
Age (yr)	71 (56–74)	67.5 (58–71)
Height (cm)	178 (170–182)	174.5 (169–180)
Weight (kg)	75.9 (10.3)	84.5 (18.5)
BMI (kg m^−2^)	25 (3)	28 (6)∗
Type of procedure		
CABG	8 (35)	8 (36)
Valve surgery	6 (26)	5 (23)
Ascending aorta surgery	1 (4)	2 (9)
Double procedure	6 (26)	5 (23)
Triple procedure	2 (9)	0
Other	0	2 (9)

**Table 2:** Primary, secondary, and other outcome variables. Data expressed as median (inter-quartile range) or number (%), unless stated otherwise. Total morphine dose, sum of nurse-administered morphine before PCA start and PCA morphine. IL-6, interleukin 6; LIDO, lidocaine group; NSAID, non-steroidal anti-inflammatory drug; PCA, patient-controlled analgesia; PEF, peak expiratory flow; POD, postoperative day; PONV, postoperative nausea and vomiting; SAL, saline group; sd, standard deviation.

	LIDO group (*n*=23)	SAL group (*n*=22)	*P*-value
Primary outcome			
Cumulative PCA morphine at 72 h (mg)	10 (5–19)	28.2 (16–42.5)	**0.014**
Secondary outcomes			
Total morphine dose at 72 h (mg)	17 (7–23)	38.2 (21–60.5)	**0.003**
Cumulative PCA morphine at 48 h (mg)	15.5 (6–23)	23 (13–33)	0.069
Total morphine dose at 48 h (mg)	20 (11–33)	33.1 (19–42)	**0.018**
Cumulative PCA morphine at 24 h (mg)	10 (3–17)	13 (5–19)	0.230
Total morphine dose at 24 h (mg)	15.5 (6–23)	23 (14–30)	**0.030**
Participants requiring oxycodone:			
2 weeks after surgery	1 (10)	5 (28)	0.375
1 month after surgery	0	2 (12)	0.492
2 and 3 months after surgery	0	0	—
PEF (L min^−1^):			
baseline	520 (363–583)	527 (457–580)	0.496
POD 1	217 (167–281)	193 (157–260)	0.530
POD 2	202 (157–278)	240 (180–280)	0.284
POD 3	200 (187–277)	260 (237–320)	**0.028**
Serum IL-6 concentration (ng L^−1^):			
baseline	2.4 (2–3.7)	2.3 (2–4.2)	0.811
1 h	59 (55–77)	50 (40–89)	0.301
24 h	130 (74–198)	110 (56–249)	0.597
48 h	113.5 (63–191)	166.5 (82–205)	0.511
72 h	65 (39–105)	83 (39–94)	0.801
Safety and other outcomes			
Participants not alert POD 1–3	14 (61)	7 (32)	0.051
Participants experiencing PONV:			
POD 0	9 (39)	8 (36)	0.848
POD 1	9 (53)	5 (26)	0.171
POD 2	7 (47)	5 (26)	0.288
POD 3	3 (27)	4 (21)	1.000
Time to tracheal extubation (min)	30 (20–50)	21 (11–55)	0.228
Length of stay, days	5 (4–7)	5 (4–7)	0.593
Perioperative fentanyl, mg (mean [sd])	1.02 (0.23)	0.96 (0.22)	0.404
Participants requiring paracetamol:			
POD 0–3	23 (100)	(22)100	
2 weeks after surgery	5 (45)	12 (67)	0.438
1 month after surgery	4 (31)	5 (29)	1.000
2 months after surgery	1 (7)	3 (17)	0.613
3 months after surgery	0	0	—
Participants requiring clonidine:			
POD 0	17 (74)	19 (86)	0.459
POD 1	0	2 (9)	0.488
POD 2	1 (5)	2 (9)	1.000
POD 3	1 (5)	0	1.000
2 weeks; 1, 2, and 3 months after surgery	0	0	—
Participants requiring NSAID:			
POD 0	3 (13)	3 (14)	1.000
POD 1	1 (5)	1 (5)	1.000
POD 2	4 (19)	2 (9)	0.412
POD 3	1 (5)	1 (5)	1.000
2 weeks; 1, 2, and 3 months after surgery	0	0	—

Bold values are below a significance level of 0.05.

### Outcomes

#### Primary outcome

At 72 h, the total PCA morphine dose was 10 mg (IQR: 5–19 mg) in the LIDO group and 28.2 mg (IQR: 16–42.5 mg) in the SAL group (*P*=0.014) (Table 2). When expressed as a weight-based dose, the difference remained statistically significant (LIDO group: 0.15 mg kg^−1^ [IQR: 0.07–0.27 mg kg^−1^], SAL group: 0.31 mg kg^−1^ [IQR: 0.21–0.53 mg kg^−1^]; *P*=0.013).

#### Secondary and safety outcomes

**Fig 2:**
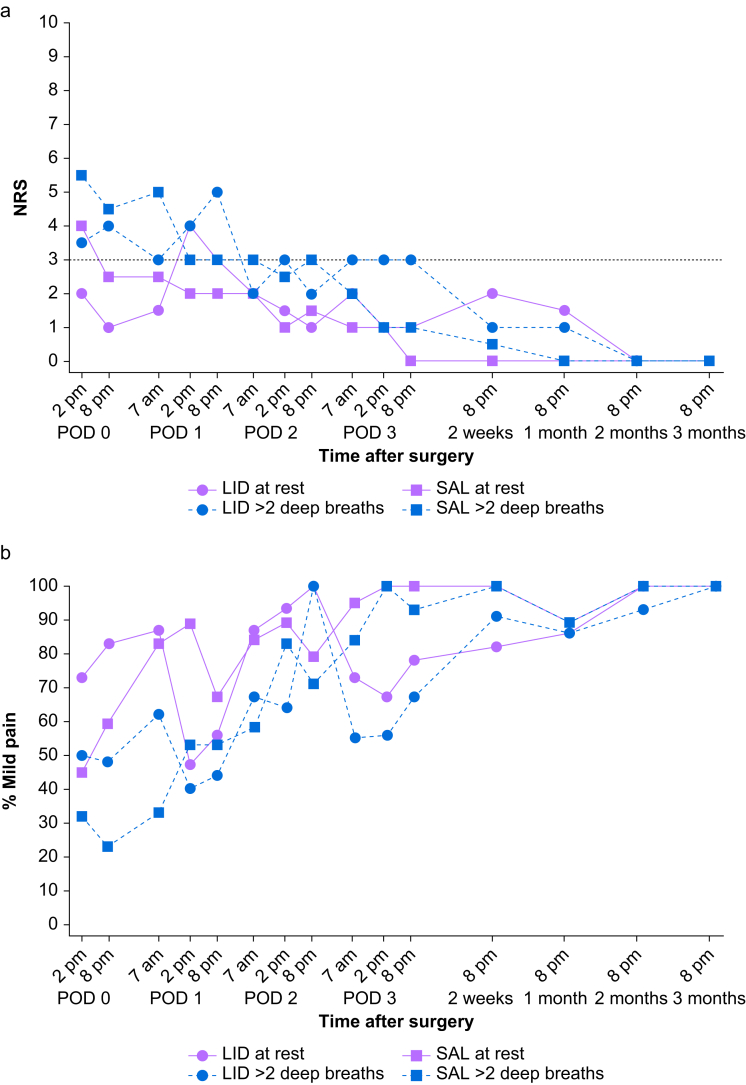
(a) Median NRS score at rest and after two deep breaths from the day of surgery until 3 months for the LIDO and SAL groups. The dashed line at NRS 3 indicates the upper limit of mild pain. (b) Percentage of participants experiencing mild pain, defined as NRS≤3, at rest and after two deep breaths from the day of surgery until 3 months for the LIDO and SAL groups. LIDO, lidocaine group; NRS, numerical rating scale; POD, postoperative day; SAL, saline group.

The total postoperative morphine dose at 24, 48, and 72 h was significantly lower in the LIDO group (Table 2). Except in the afternoon of POD 0 for the SAL group and in the afternoon of POD 1 for the LIDO group, the median NRS was ≤3 during the entire follow-up period (Fig. 2a). The median NRS after two deep breaths was >3 in the afternoon and evening of POD 0 for both treatment groups. At POD 1, the NRS was between 3 and 5, without differences between treatment groups. From POD 2 and the remainder of the follow-up, the median NRS after two deep breaths was ≤3.

A larger proportion of participants in the LIDO group experienced mild pain both at rest and after two deep breaths at POD 0 (Fig. 2b). This is in contrast to the afternoon and evening of POD 1, when a larger percentage of the SAL group experienced mild pain at rest. From POD 2 onwards, the percentage of participants with mild pain at rest and after two deep breaths had a similar course for both treatment groups. At 3 months, all participants experienced mild pain.

Table 2 shows the results for the secondary, safety, and other outcomes. PEF was significantly higher in the SAL group at POD 3. Serum IL-6 concentrations, the presence of alertness, incidence of PONV, time to tracheal extubation, and length of stay did not differ between treatment groups.

At 2 weeks after surgery, a total of six participants required oral oxycodone treatment and this had reduced to two patients at 1 month after surgery, with no significant difference between the treatment groups. No participant required opioid analgesia at 2 or 3 months after surgery (Table 2).

**Fig 3:**
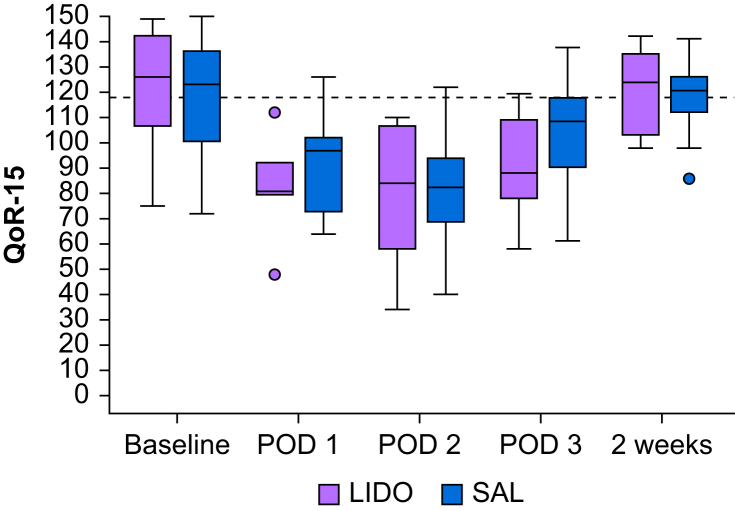
QoR-15 scores from preoperative values (baseline) to 2 weeks after surgery. The QoR-15 score ranges from 0 to 150, with a minimal clinically important difference of 6.0 and patient acceptable symptom state of 118,^22^^,^^26^ indicated by the dashed line. Boxes: upper hinge represents 75th percentile, lower hinge represents 25th percentile, central line represents median, and whiskers represent minimum and maximum values excluding outliers. Circles represent outliers, defined as at least 1.5 times the inter-quartile range. SAL group QoR-15 from POD 2 to POD 3, *P*=0.028. LIDO, lidocaine; POD, postoperative day; QoR-15, Quality of Recovery-15; SAL, saline.

There was no significant difference in QoR-15 score between the groups at any time from the evening before surgery until 3 months after surgery (Fig. 3). The lowest score was seen at POD 2, 84 (IQR, 58–107) for the LIDO group and 82 (IQR, 69–94) for the SAL group. The improvement in QoR-15 score from POD 2 to 3 was significant for the SAL group only (POD 2: 82 [IQR, 69–94]; POD 3: 108.5 [IQR 90–118]; *P*=0.028). Both groups returned to preoperative QoR-15 levels at 2 weeks after surgery, with a score >118.

**Fig 4:**
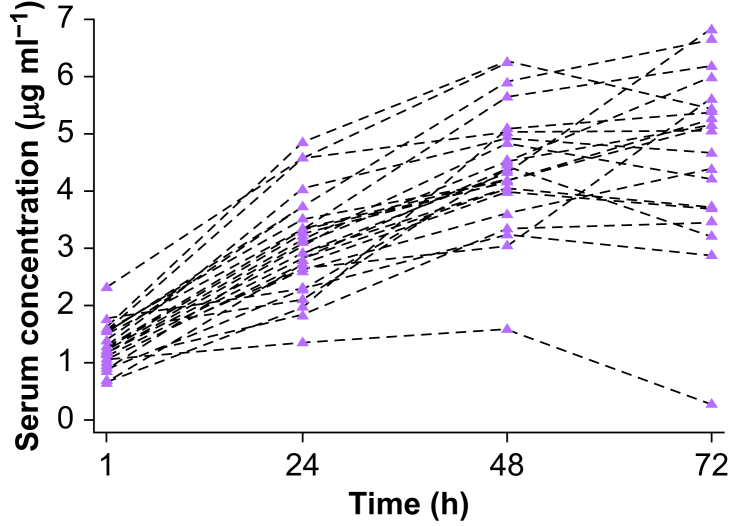
Serum lidocaine concentration over time for individual participants in the LIDO group. The first sample was obtained 1 h after the administration of the lidocaine bolus and the initiation of the infusion. LIDO, lidocaine.

In the LIDO group, lidocaine serum concentrations increased from 1 to 24 h, and again to 48 h (Fig. 4). However, from 48 to 72 h, the serum concentration increased in 10 participants, remained unchanged in two, and decreased in eight. Twelve participants had at some point a serum lidocaine concentration >5 μg ml^−1^, the upper limit of the therapeutic interval for treatment of ventricular arrhythmias.^18^ In a *post hoc* analysis, we categorised participants into groups with serum lidocaine concentrations <5 respectively >5 μg ml^−1^. Group comparison revealed no difference in alertness. There was no difference in the incidence of arrhythmia between groups (details are provided in the Supplementary material).

During the 12-week follow-up, 16 participants had 24 SAEs without difference between treatment groups (Supplementary Table S1).

## Discussion

After sternotomy for open heart surgery, a 72-h bilateral parasternal block with lidocaine that was initiated after wound closure reduced the PCA morphine requirement from 28.2 to 10.0 mg. Including nurse-administered morphine given before initiation of the PCA pump, total morphine requirement was significantly lower than in the SAL group at 24, 48, and 72 h. Pain for both the LIDO and SAL groups was well controlled overall. Pain at rest and after two deep breaths on POD 0 and 1 was mild to moderate, thereafter mild for the remainder of the follow-up. From POD 2 a similar increase in the percentage of participants experiencing mild pain was seen. This confirms participants received adequate multimodal analgesia irrespective of treatment group.

The decrease in morphine requirement for the LIDO group is comparable to the most pronounced opioid reduction found in previous trials that infused ropivacaine or bupivacaine.^5^ ^6^ ^8^ ^9^ ^11^ Our interpretation is that lidocaine for a parasternal block was effective, but that the high dose used did not appear to result in a further improvement of effect, although we did not directly compare local anaesthetics in this trial. This is in contrast to findings from a meta-regression that showed that paravertebral blocks with a higher than recommended dose of bupivacaine had an improved effect.^24^ The IL-6 concentrations we found were comparable to previous findings after cardiac surgery.^25^ However, in contrast to the results from a recent meta-analysis,^16^ lidocaine did not result in a decrease in serum IL-6 concentrations for the LIDO group despite achieving an adequate serum concentration.^23^ This could be because the lidocaine infusion was started after the end of surgery, which may be too late for the anti-inflammatory effect to be observed.

Recovery as assessed by the QoR-15 score did not differ between treatment groups at any time during the 3-month follow-up. Both groups reached a QoR-15 score of >118 at 2 weeks, which indicates an acceptable recovery.^26^ The increase of 16.5 points in QoR-15 score between POD 2 and 3 for the SAL group was statistically significant and more than double the defined minimal clinically important difference (MCID).^26^ ^27^ None of the other secondary outcomes indicative of postoperative wellbeing, such as alertness, PONV incidence, or length of stay, showed any difference between groups. The clinical significance of the SAL group's higher PEF value at POD 3 is uncertain. To our knowledge an MCID for PEF has not been established. The difference could be a result of the limited analgesic effect of the parasternal block. Concurrent with the reduced PEF, the LIDO group had a decline in the proportion of participants who experienced mild pain after two deep breaths. Other parts of the chest can cause pain after sternotomy^28^ ^29^ and exhaling with force as required for PEF measurement could have been hampered by pain that was neither attenuated by the block, nor morphine.

We did not find any difference in secondary outcomes or adverse events between treatment groups. Because of the small number of participants in this trial and the exploratory nature of the secondary outcomes, no firm conclusions can be drawn. However, if several of the secondary outcomes and the lower response rates for the NRS and QoR-15 scores during POD 0–3 are considered together, it seems that the LIDO group could have had an overall inferior recovery compared with the SAL group. We can see two possible explanations. First, we cannot rule out a mild lidocaine toxicity. Some of the serum lidocaine concentrations were close to potential CNS toxic levels. We did not analyse the serum concentration of lidocaine's active metabolites, monoethylglycinexylidide and glycinexylidide, and therefore, it is possible that these contributed to a mild toxic effect in some participants. Although no overt toxic symptoms were reported, the combination of the low NRS and QoR-15 score response rates and the larger number of participants in the LIDO group who were not alert at POD 1 and 2, could indicate an effect of lidocaine on the CNS. However, it is not unusual for cognition to be affected after cardiac surgery, and this is an alternative explanation for our findings.

### Limitations

This was a small single-centre trial. Although we demonstrated a statistically significant difference in the primary outcome, its clinical relevance is uncertain: the other results should be interpreted with caution.

The trial had missing data. We were dependent on participants responding to push notices on their mobile phones and actively opening a link to participate in the follow-up. In the evenings of POD 1, 2, and 3, only one-third of participants in the LIDO group returned QoR-15 scores, compared with two-thirds of participants in the SAL group.

In addition to PCA morphine, several other non-opioid analgesics were administered. The practice of multimodal analgesia is a reality in the clinical setting of this trial. If deemed necessary to achieve acceptable analgesia, NSAID and clonidine were used. There was no difference in the use of these medications between treatment groups.

### Conclusions

After sternotomy for open heart surgery and compared with standard care, a 72-h bilateral parasternal infusion of lidocaine started at the end of surgery resulted in a statistically significant decrease in the total postoperative morphine requirement from 38.2 to 17.0 mg. Pain was well controlled in both the LIDO group and the SAL group. No difference in secondary outcomes pertaining to the inflammatory response nor recovery was found. Considering benefit *vs* potential harm, we cannot recommend a 72-h bilateral parasternal lidocaine infusion after cardiac surgery. Standard multimodal analgesic treatment provided adequate pain treatment and resulted in at least equal recovery.

Considering that pain and opioid requirement were reduced during the first 24 h for the group that received active treatment, the further exploration of peripheral thoracic nerve blocks as part of a multimodal analgesic strategy seems warranted. The timing, dose, and duration of the infusion with regard to both block efficacy and risk of local anaesthetic toxicity need further study, and the effect on the overall intermediate recovery, including cognitive state.

## Authors’ contributions

Academic sponsor: JJ.

Principal investigator: ML.

Study design: ML, AÖ, JJ.

Data analysis and interpretation: all authors.

Drafting of the manuscript: ML.

Critical revision of the manuscript for important intellectual content: all authors.

## Funding

The Function Perioperative Medicine and Intensive Care, Section for Cardiothoracic Anaesthesia and Intensive Care and the Cardiovascular Surgical Ward at the Karolinska University Hospital in Stockholm. A non-restricted grant in support of this trial from Grünenthal Sweden AB to ML (Karolinska Institute's registration no 2–2191/2020).

The Swedish Heart-Lung Foundation (20190533 to US). AFC is supported by an independent donation from Mr Fredrik Lundberg. The funders have had no role in study design, data collection and analysis, preparation of the manuscript, nor the decision to publish.

## Declarations of interest

JJ is a paid consultant safety physician at AstraZeneca. The other authors declare that they have no conflicts of interest.
