# Supplementary material for: The effect of continuous bilateral parasternal block with lidocaine on patient-controlled analgesia opioid requirement and recovery after open heart surgery: a double-blind randomised controlled trial
Source: BJA Open. 2024 Apr 20;10:100279. doi: 10.1016/j.bjao.2024.100279 (PMC11046074; doi:10.1016/j.bjao.2024.100279)
Supplement: Multimedia component 1 [file mmc1.docx]

A continuous bilateral parasternal block with lidocaine reduces patient-controlled analgesia opioid requirement after open heart surgery but does not affect recovery: a double-blind randomised controlled trial

SUPPLEMENTARY MATERIAL

**Methods**

Intervention

Except for the allocated treatment and the use of a patient-controlled analgesia (PCA) pump, all study participants received care according to standard department practice. Prior to surgery, participants were informed how to use an intravenous PCA pump, how to score pain according to the numeric rating scale (NRS), and how to access and use the eHealth platform for the postoperative 3-month follow-up.

Consistent to department practice, participants were provided anaesthesia with sevoflurane (minimal alveolar concentration (MAC) of 1.0) before as well as after, and propofol infusion during extracorporal circulation (ECC). The study’s standard operating procedure (SOP) protocol recommended a perioperative fentanyl dose of 0.015 – 0.02 mg.kg^-1^ and administration of intravenous (iv) morphine at 0.1 mg.kg^-1^ as well as 1000 mg of paracetamol after separation from cardiopulmonary bypass (CPB). Dosing of anaesthetics and analgesics was at the attending anaesthesiologist’s discretion.

*Procedure for regional anaesthesia*

At the end of the procedure, after wound suturing, the skin surrounding the sternal wound was swabbed with chlorhexidine. The surgeon then percutaneously inserted a multi-hole 19-cm silver-coated catheter under the pectoral muscle (ON-Q Soaker; Avanos Medical, Alpharetta, GA) on either side of the sternum, positioning it over the costosternal margin. A tunnelling stylet covered by a sheath was inserted percutaneously 2-3 cm lateral and 2-3 cm inferior to the xyphoid process. Subpectorally and parallel to the sternum, the stylet was carefully advanced in a cephalad direction to a position cranial to the sternal incision. After removal of the stylet and insertion of the catheter, the sheath was peeled back and removed.

*Start of the intervention*

Depending on allocation, a 20-ml bolus of either lidocaine 5 mg.ml^-1^ (LIDO group) or saline 9 mg.ml^-1^ (0.9%) (SAL group) was administered in each catheter. Both catheters were then connected to a single-use elastomeric pump containing either lidocaine at 5 mg.ml^-1^ (LIDO group) or saline (SAL group). The infusion rate of 7 ml.h^-1^ resulted in an hourly lidocaine dose of 70 mg and daily dose of 1680 mg. The elastomeric pump containing the allocated treatment was replaced by a new after 24 and 48 hours, for a total treatment duration of 72 hours.

*Postoperative care and assessment*

After transfer to our Thoracic Intensive Care and Recovery Unit participants were extubated in accordance with the department’s fast track protocol. If more than mild pain (NRS >3) was experienced, the rescue analgesic regimen was as follows: intravenous (iv) boluses of morphine at 1-mg increments up to a total dose of 0.15 mg.kg^-1^, iv ketorolac at 15 – 30 mg if NSAID was tolerated and deemed acceptable regarding chest tube flow and/or iv clonidine at 15 – 75-μg increments if tolerated with regard to alertness and blood pressure. Once mild or no pain (NRS score ≤ 3) was achieved, and the participant was alert, a PCA pump was connected for the duration of the intervention, allowing for self-administration of intravenous morphine at 1 mg boluses, with a lockout time of 6 minutes and a maximal dose of 30 mg every 4 hours. Instructions were to use the PCA pump if pain was more than mild (NRS > 3). All participants received oral paracetamol at 1 g four times daily. If necessary, the same rescue analgesic regimen as above could be used, with the added possibility of oral NSAID treatment, consisting of naproxen 250-500mg twice daily. After conclusion of the intervention, the parasternal catheters were removed, the PCA pump discontinued and an oral opioid regimen consisting of slow-release oxycodon at an equivalent dose to the PCA morphine requirement of the last 24 hours was initiated. Oral paracetamol and, if initiated earlier, NSAID was continued.

At 3, 6 and 9 hours after extubation, participants were asked to score their pain according to NRS, both at rest and after two deep breaths. No pain was to be scored as 0, mild pain as 1–3, moderate pain as 4–6, and severe pain as 7–10. At the same time, symptoms of postoperative nausea and vomiting (PONV) and degree of sedation according to the Richmond Agitation-Sedation Scale were evaluated.

Preoperatively and in the afternoon of postoperative days (PODs) 1-3 participants performed three consecutive peak expiratory flow (PEF) measurements. At the same visit, participants were evaluated for sedation with a dichotomous score of “alert” versus “other than alert”. Two blood samples were drawn before surgery and at 1, 24, 48 and 72 hours after the start of the intervention. The first sample was analysed imediately for IL-6 concentration. The second sample was centrifuged at 2000 g for 10 minutes, after which the serum was pipetted into micro tubes and stored at -70°C. After the study was concluded, serum lidocaine concentrations were analysed. During the entire intervention, participants were continuously monitored for the occurrence of arrhythmias.

From the evening prior to surgery, study participants received push notices at predetermined follow-up times on their mobile phone. The push notices contained a link to a time-specific questionnaire on the eHealth platform. After two-way identification, access was gained. Participants were asked to complete the following: (1) Quality of Recovery(QoR)-15 score the evening before surgery, in the evening of POD1-3, as well as at 2 weeks, and 1, 2 and 3 months; (2) pain according to the NRS at rest and after two deep breaths in the evening of POD 0, in the morning, afternoon, and evening of POD 1 – 3, as well as at 2 weeks, and 1, 2 and 3 months; (3) in the evening of the day of surgery, in the morning, afternoon, and evening of POD 1 – 3, as well as at 2 weeks, and 1, 2 and 3 months.

During the intervention and the 3-month follow-up participants were monitored for any adverse event.

Randomisation

Treatment was randomised in a 1:1 fashion in blocks of eight, except for the last block of five. Before the trial, sequentially numbered opaque envelopes were prepared with the allocated treatment randomly distributed in each block. At enrolment, each trial participant received an inclusion number. After surgery start, one of two nurse anaesthetists who were otherwise not involved in the trial opened the envelope with the number corresponding to the participant’s inclusion number. Based on allocation, three elastomeric pumps prepared in advance by the hospital pharmacy were selected, blinded and together with two blinded 20-ml syringes containing the corresponding treatment delivered to the team caring for the trial participant.

**Results**

Missing data

Figure 1 presents the percentage of participants that did not complete NRS or QoR-15 score during the 3-month follow-up.

PEF

Figure 2 presents the PEF value from baseline to POD 3 for the LIDO and SAL group.

Safety and adverse events (Table 1)

New onset atrial fibrillation occurred in four participants in the LIDO group (17%) and in six (27%) in the SAL group. The incidence of sustained bradycardia did not differ between groups either, affecting five respectively three participants in the LIDO and SAL group. In total 4 episodes of non-sustained ventricular tachycardia (NS-VT) were recorded in four of the participants in the LIDO group, and 11 episodes in five of the participants in the SAL group. No ventricular fibrillation occurred.

During the 12-week follow-up, two participants in the LIDO group and three participants in the SAL group were in need of a permanent pacemaker.

*Serious adverse events*

Participants were monitored for serious adverse event (SAE) until 3 months after surgery. There was no difference in SAE between treatment groups (Table 1).

*
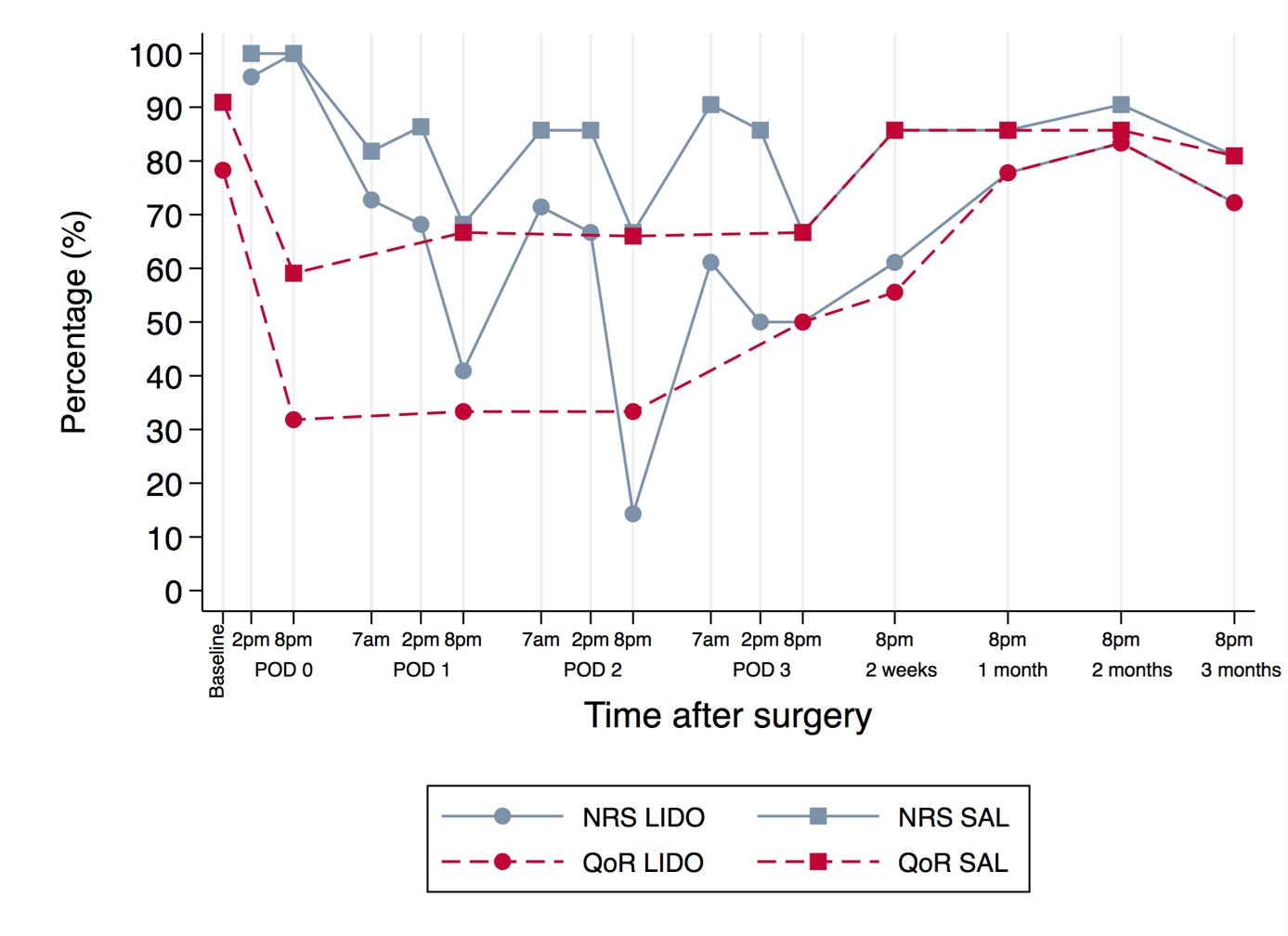
*

**Figure 1.** Percentage of participants in the LIDO and SAL groups that returned NRS and QoR-15 scores at the different time points after surgery. Baseline, preoperative score; POD, postoperative day; QoR-15, Quality of Recovery-15 ; LIDO, lidocaine group; SAL, saline group

**
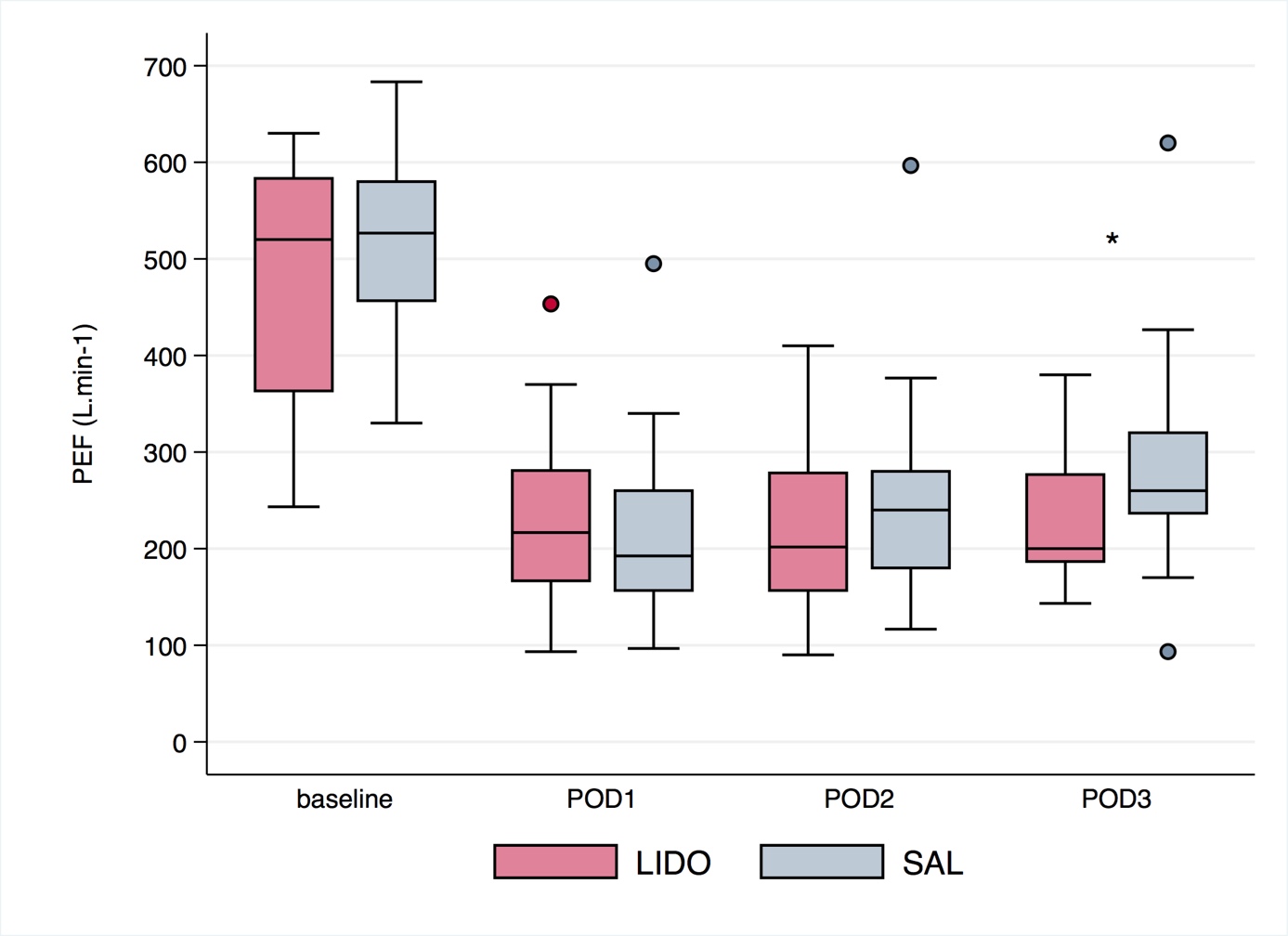
**

**Figure 2.** PEF from preoperative values (baseline) to POD3. Boxes: upper hinge represents 75^th^ percentile, lower hinge 25^th^ percentile, central line the median, whiskers minimum and maximum values excluding outliers. Circles represent outliers, defined as at least 1.5 times the interquartile range. PEF, peak expiratory flow; POD, postoperative day; SAL, saline; LIDO, lidocaine; *, *p* = 0.028

**Table 1.** Serious adverse events until 3 months after surgery. Data are presented as number of SAE that occurred, and number (%) of participants affected. SAL, saline; LIDO, lidocaine; SAE, serious adverse event; UTI, urinary tract infection

| **SAE** | **Overall (n=45)** | **LIDO (n=23)** | **SAL (n=22)** |
| --- | --- | --- | --- |
| Redo bleeding | 4 | 1 | 3 |
| Redo tamponade | 1 | 1 | 0 |
| Seizure | 1 | 0 | 1 |
| Respiratory failure | 3 | 3 | 0 |
| Wound infection | 2 | 1 | 1 |
| Mediastinitis | 1 | 1 | 0 |
| Pneumonia | 1 | 1 | 0 |
| UTI | 2 | 1 | 1 |
| Stroke | 1 | 1 | 0 |
| Permanent pacemaker | 5 | 2 | 3 |
| Ulcus | 3 | 2 | 1 |
| Any SAE | 16 (36%) | 9 (39%) | 7 (32%) |
| One SAE | 10 (22%) | 5 (22%) | 5 (23%) |
| Two SAE | 5 (11%) | 3 (13%) | 2 (9%) |
| Four SAE | 1 (2%) | 1 (4%) | 0 |
